# Intranasal administration of a recombinant RBD vaccine induces long-term immunity against Omicron-included SARS-CoV-2 variants

**DOI:** 10.1038/s41392-022-01002-1

**Published:** 2022-05-17

**Authors:** Hong Lei, Aqu Alu, Jingyun Yang, Wenyan Ren, Cai He, Tianxia Lan, Xuemei He, Li Yang, Jiong Li, Zhenling Wang, Xiangrong Song, Wei Wang, Guangwen Lu, Xiawei Wei

**Affiliations:** 1 Laboratory of Aging Research and Cancer Drug Target, State Key Laboratory of Biotherapy and Cancer Center, National Clinical Research Center for Geriatrics, West China Hospital, Sichuan University, 610041 Chengdu, China; 2 WestVac Biopharma Co. Ltd., Chengdu, China

**Keywords:** Vaccines, Senescence

## Abstract

The outbreak of coronavirus disease 2019 (COVID-19) has posed great threats to global health and economy. Several effective vaccines are available now, but additional booster immunization is required to retain or increase the immune responses owing to waning immunity and the emergency of new variant strains. The deficiency of intramuscularly delivered vaccines to induce mucosal immunity urged the development of mucosal vaccines. Here, we developed an adjuvanted intranasal RBD vaccine and monitored its long-term immunogenicity against both wild-type and mutant strains of severe acute respiratory syndrome coronavirus-2 (SARS-CoV-2), including Omicron variants, in mice. Three-dose intranasal immunization with this vaccine induced and maintained high levels of neutralizing IgG antibodies in the sera for at least 1 year. Strong mucosal immunity was also provoked, including mucosal secretory IgA and lung-resident memory T cells (T_RM_). We also demonstrated that the long-term persistence of lung T_RM_ cells is a consequence of local T-cell proliferation, rather than T-cell migration from lymph nodes. Our data suggested that the adjuvanted intranasal RBD vaccine is a promising vaccine candidate to establish robust, long-lasting, and broad protective immunity against SARS-CoV-2 both systemically and locally.

## Introduction

The ongoing coronavirus disease 19 (COVID-19) pandemic is triggering a global health crisis accounting for over 500 million confirmed cases and 6 million deaths at the time of writing. (WHO, 2022, https://covid19.who.int/). As the pathogen of COVID-19, severe acute respiratory syndrome coronavirus-2 (SARS-CoV-2) attacks human cells by binding to cell entry receptor angiotensin-converting enzyme II (ACE2) with its receptor-binding domain (RBD) on spike protein.^1–4^ ACE2 receptors are mainly expressed on cells of the respiratory tracts, which makes SARS-CoV-2 a mucosal pathogen and COVID-19 a mucosal transmitted disease.^5^ In an unprecedented speed, hundreds of vaccines have been developed and evaluated in preclinical models or clinical trials, with 38 candidates approved for emergency use in humans (WHO, 2022, https://www.who.int/emergencies/diseases/novel-coronavirus-2019/covid-19-vaccines). Among different vaccine types, recombinant protein vaccines exhibit superior safety profiles and high immunogenicity in the presence of proper adjuvants.^6^

The currently licensed vaccines demonstrated efficacy and tolerability against SARS-CoV-2 infections, including the severe cases.^7–10^ However, the established antiviral immunity was observed to wane or diminish in several months post-immunization, increasing the risk of breakthrough infection.^11–14^ Humoral immune responses were substantially decreased six months after immunization with BNT162b2 vaccine, the firstly licensed mRNA vaccine of SARS-CoV-2.^12^ Similarly, recent studies reported that the neutralizing antibody (NAb) responses could only last for about 2 to 3 months in most patients recovering from SARS-CoV-2 infection, making them vulnerable to re-inifection.^15–17^ Therefore, an additional booster may be required beyond the original vaccination schedule, causing a waste of time, money, and resources. In addition, the emergence of various mutant strains of SARS-CoV-2, including B.1.1.7 (Alpha), B.1.351 (Beta), P.1 (Gamma), B.1.617.2 (Delta), C.37 (Lambda), and B.1.1.529 (Omicron) strains, may cause immune evasion and fold changes in the effectiveness of the currently licensed vaccines.^18–21^ Therefore, it is of vital importance to develop vaccines that could induce long-term and broad immune responses against the existent and future variant strains of SARS-CoV-2.

All the currently licensed COVID-19 vaccines belong to injected vaccines, which are strong inducers of systemic immune responses but poor in evoking mucosal immunity. In intramuscularly immunized animals, absence of mucosal immunity resulted in viral replication and persistent nasal shedding in the upper respiratory tracts when challenged with live SARS-CoV-2.^22,23^ Mucosal immunity is dominantly composed of secretory IgA (sIgA) and tissue-resident memory T (T_RM_) cells. In its polymeric form, sIgA can neutralize invading viral particles at the site of infection.^24^ T_RM_ cells, which express high levels of resident markers including integrin α_E_ (CD103) and C-type lectin (CD69), are localized at barrier tissues and can respond immediately after secondary infections.^25^ Lung CD8^+^ T_RM_ cells can prevent respiratory viral infection partly through rapid interferon-γ (IFN-γ) production.^26^ Relatively, lung CD4^+^ T_RM_ cells are essential for the formation of lung CD8^+^ T_RM_ cells and the induction of humoral responses in the lungs.^27,28^ Moreover, vaccine-generated lung T_RM_ cells can provide cross-protection against pulmonary virus infection.^29,30^ At present, a few intranasal COVID-19 vaccines have been investigated in preclinical studies, which could induce strong systemic and mucosal immune responses.^31^ 12 candidates have reached clinical trials of different phases, including virus-vectored vaccines, live attenuated vaccines, and protein subunit vaccines.^31^ However, none of the studies reported the long-term immunogenicity of the intranasal vaccines up to 1 year.

Previously, our team developed an intranasally administered recombinant RBD vaccine that induced robust humoral and cellular immunity against SARS-CoV-2 in the presence of polyethyleneimine (PEI) adjuvant in mice.^32^ Here, we monitored the long-term immunogenicity and safety of the same vaccine. We demonstrated that 1 year after vaccination, the adjuvanted RBD vaccine induced and maintained high levels of humoral and mucosal immunity, characterized by systemic neutralizing IgG antibodies, mucosal IgA antibodies, and local T-cell immunity in the lungs. Substantial neutralizing of SARS-CoV-2 variants of concern (VOCs) was also assessed, including B.1.1.7, B.1.351, P.1, B.1.617.2, and the most recent Omicron variants. Mechanism studies indicated that the maintenance of lung T_RM_ cells largely depends on local T-cell proliferation, instead of T-cell migration from lymph nodes. The promising results supported further development of this vaccine against current and future variant strains of SARS-CoV-2.

## Results

### Intranasal vaccination with adjuvanted RBD vaccine induces broad and long-lasting humoral immune responses against SARS-CoV-2, including Omicron variant

**Fig. 1:**
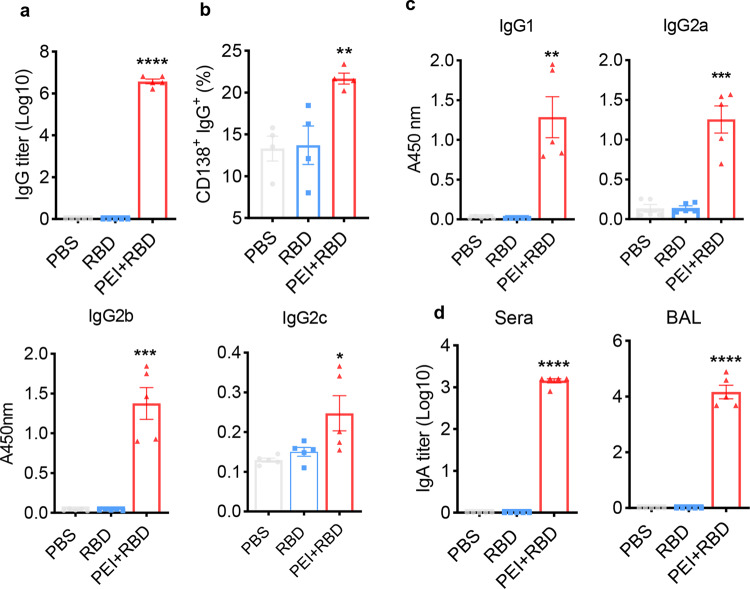
Humoral responses in 1 year after intranasal immunization with RBD vaccine. NIH mice were intranasally immunized with PBS, RBD or PEI-adjuvanted RBD on days 0, 7, and 21. On day 365 after the first dose, RBD-specific antibodies in the serum and BALs were determined with ELISA. **a** RBD-specific total IgG levels in the sera of immunized mice. **b** Frequency of IgG-secreting CD138^+^ long-lived plasma B cells in bone marrow of immunized animals on day 365, as determined by FCM. **c** Further analysis of RBD-specific IgG isotypes in the serum, including IgG1, IgG2a, IgG2b, and IgG2c. **d** RBD-specific IgA titers in the sera and BALs of immunized animals on day 365. All the data were presented as mean ± SEM. *n* = 4–5. *P* values were compared to PBS group (*P < 0.05; **P < 0.01; ***P < 0.001; ****P < 0.0001)

To evaluate the long-term immunogenicity of the RBD vaccine, groups of NIH mice (*n* = 5 per group) were immunized with PBS, RBD alone or RBD with PEI adjuvant on days 0, 7, 21 via intranasal drops. 365 days after the prime immunization, serum samples were collected to assess the humoral immune responses. The ELISA results indicated that intranasal immunization with PEI-adjuvanted RBD, but not PBS or RBD alone, induced high levels of long-lasting RBD-specific IgG antibodies in sera (Fig. 1a). The endpoint titer (Log10) of serum IgG was 6, comparable to that observed on day 35 as previously reported by our team (6.114, *P* = 0.074).^32^ The long-term increase in serum IgG titers is correlated with an increase in IgG-secreting long-lived plasma cells (CD138^+^) in the bone marrow (Fig. 1b), which can constitutively produce high levels of antibody.^33^ IgG subtype analysis indicated that IgG1 (Th2-associated isotype^34^), as well as IgG2a, IgG2b, and IgG2c (Th1-associated isotype^35^) levels were all increased after immunization with the PEI-adjuvanted RBD vaccine (Fig. 1c). We also evaluated SARS-CoV-2-specific IgA responses in sera and bronchoalveolar lavage fluids (BALs) of the immunized mice. Immunization with PEI + RBD, rather than PBS or pure RBD, elicited high levels of RBD-specific IgA antibodies in sera and BAL on day 365 (Fig. 1d), indicating the induction of potent and enduring mucosal immunity.

**Fig. 2:**
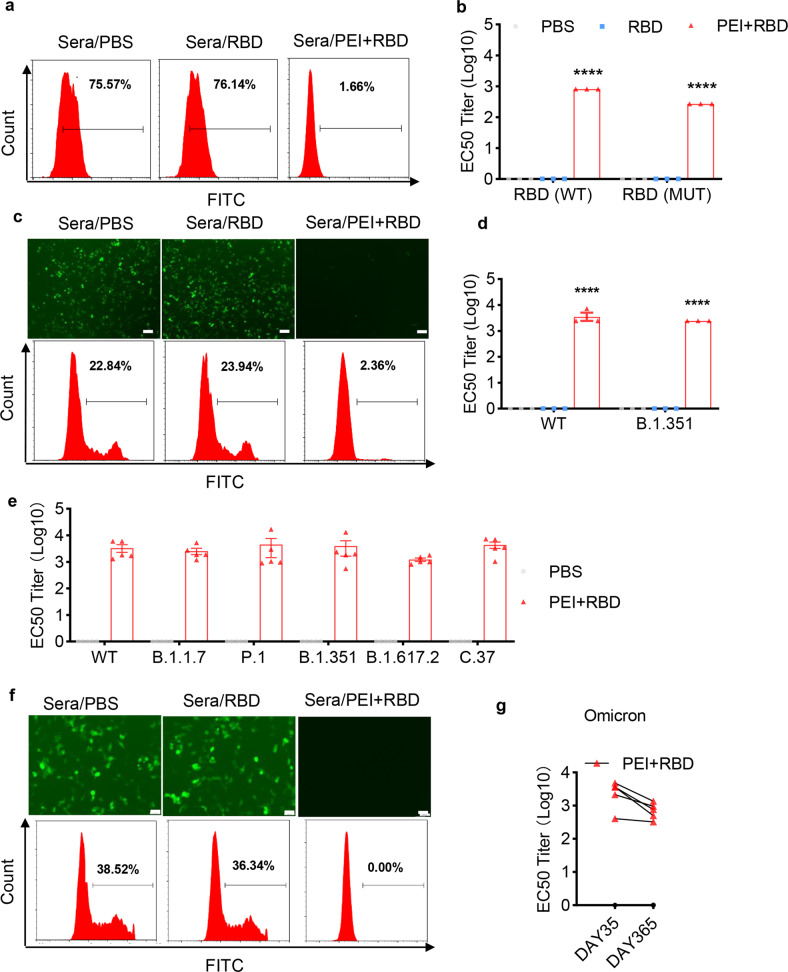
Long-term functional characterization of the immune sera from mice intranasally immunized with RBD vaccine. NIH mice were intranasally immunized with PBS, RBD or PEI-adjuvanted RBD on days 0, 7, and 21. Immune sera were collected on day 365 for functional characterization. **a**, **b** Blockade of WT or mutated RBD’s binding to cell surface ACE2 receptors by immune sera was assessed with FCM. RBD-MUT indicated three mutations (K417N, E484K and N501Y) located in the RBD. **c**, **d** Long-term immune sera neutralized the infection of EGFP-expressing SARS-CoV-2 pseudovirus to 293T/ACE2 cells as determined by FCM and fluorescent microscopy., including both WT and B1.351 variant pseudoviruses. Scale bar, 100 μm. **a** and **c** were performed at 1:270 dilution. **e** Long-term immune sera neutralized the infection of luciferase-expressing SARS-CoV-2 pseudovirus to 293 T/ACE2 cells, including WT, B.1.1.7, P.1, B.1.351, B.1.617.2, and Lambda variant pseudoviruses. Long-term immune sera neutralized the infection of Omicron variant pseudoviruses to 293T/ACE2 cells, as determined with EGFP-expressing (**f**) or luciferase-expressing (**g**) pseudoviruses. Scale bar, 100 μm. Neutralization EC50 titer is defined as the inverse dilution that achieved 50% neutralization. All the data were presented as mean ± SEM. *n* = 3 or 5. *P* values were compared to PBS group (****P < 0.0001)

For functional characterization, immune sera collected on day 365 were used to block the specific binding between RBD and ACE2 receptors. Immune sera from PEI + RBD-immunized mice effectively decreased wild-type (WT) RBD-ACE2 binding from over 70% to less than 5% at 1:270 dilution (Fig. 2a), showing 50% inhibition at titer (EC50 titer, Log10) of 2.9 (Fig. 2b). Notably, antibodies from mice immunized with PEI + RBD also remarkably inhibited the binding of a mutated RBD (RBD-MUT, including K417N, E484K, N501Y mutations) to ACE2 receptors (Fig. 2b), suggesting its potential protectivity against variant strains of SARS-CoV-2. We next evaluated the long-term neutralizing activity of the immune sera by using enhanced green fluorescent protein (EGFP)-expressing pseudoviruses to infect 293T cells that stably express ACE2 (293T/ACE2). We observed that immune sera from PEI + RBD group significantly protected 293T/ACE2 cells from WT pseudovirus infection, as characterized by decreased number of EGFP-positive cells using fluorescence microscopy and flow cytometry (FCM) (Fig. 2c, d). In addition, the immune sera showed equivalent potency in neutralizing B.1.351 variant pseudovirus with a neutralizing EC50 (neutralizing 50% of pseudovirus, Log10) of 3.39 (Fig. 2d). We verified these results using a luciferase assay and further proved the broad protectivity of the immune sear by neutralizing other common mutant strains of SARS-CoV-2, including B.1.1.7, P.1, B.1.617.2, and C.37 (Fig. 2e).

Omicron variant is a recently reported mutant strain of SARS-CoV-2, which exhibit much more mutations in the spike protein than previously evolved variants.^36^ These profiles raise concerns about enhanced transmissibility and immune evasion. In our study, we discovered that the humoral immune responses triggered by PEI + RBD immunization effectively neutralized the infection of Omicron pseudovirus on day 365 (Fig. 2f). Moreover, the protectivity only exhibited a slight decrease when compared to that detected on day 35 (Fig. 2g). Together, these data demonstrated that intranasal vaccination with the PEI-adjuvanted RBD vaccine can induce robust, broad, and long-lasting antibody responses against not only WT, and also multiple variant strains of SARS-CoV-2 both locally and systemically.

### Intranasal vaccination with adjuvanted RBD vaccine elicits strong and long-lasting lung T_RM_ cell responses

**Fig. 3:**
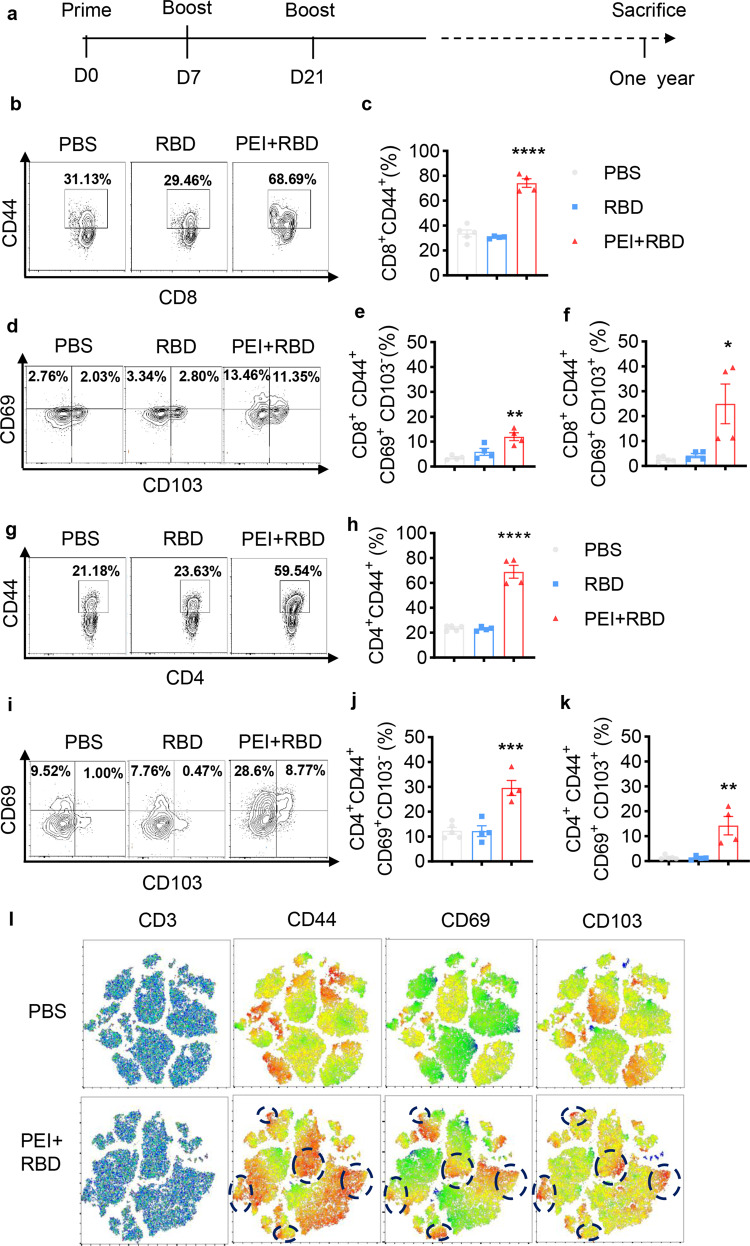
Intranasal immunization with adjuvanted RBD vaccine induces long-lasting lung T_RM_ immune responses. **a** NIH mice were intranasally immunized with PBS, RBD or PEI-adjuvanted RBD on days 0, 7, and 21. Immunized mice were sacrificed in 1 year after prime immunization and lung tissues were harvested. Lung T-cell responses were evaluated with FCM. Representative FCM plots (**b**) and quantification (**c**) of CD8^+^CD44^+^ T cells in the lungs of immunized mice. (**d**) Representative FCM plots of CD69 and CD103 expression on CD8^+^CD44^+^ T cells in the lungs. Quantification of lung CD8^+^CD44^+^CD69^+^CD103^-^− (**e**) and CD8^+^CD44^+^CD69^+^CD103^+^ (**f**) T cells. Representative FCM plots (**g**) and quantification (**h**) of CD4^+^CD44^+^ T cells in the lungs of immunized mice. **i** Representative FCM plots of CD69 and CD103 expression on CD4^+^CD44^+^ T cells in the lungs. Quantification of lung CD4^+^CD44^+^CD69^+^CD103^−^ (**j**) and CD4^+^CD44^+^CD69^+^CD103^+^ (**k**) T cells. **l** t-SNE maps were generated from CD3^+^ gated lung cells in immunized mice (3/group) and heatmap projections of CD44, CD69 or CD103 were showed on t-SNE maps. Hashed circles indicate T_RM_ cells in the lungs. Flowjo software (V.10) was used to analyze the FCM data. All the data were presented as mean ± SEM. *n* = 4–5. *P* values were compared to PBS group (*P < 0.05; **P < 0.01; ***P < 0.001; ****P < 0.0001)

To assess T-cell responses in the lungs, mice intranasally immunized with PBS, RBD alone, or PEI-adjuvanted RBD were sacrificed in 1 year after the first vaccination (Fig. 3a). Lungs were harvested and T cells were analyzed with FCM. The gating strategy for lung T cells is shown in Supplementary Fig. 1. We observed a marked increase in the frequency of memory CD8^+^ T cells (CD8^+^ CD44^+^) in the lungs of mice that received PEI-adjuvanted RBD vaccine than mice vaccinated with PBS or RBD (Fig. 3b, c). Memory CD8^+^ T cells in the lungs after PEI + RBD immunization expressed substantially higher levels of tissue-resident markers CD69, CD103, or both, while memory CD8^+^ T cells stimulated by PBS or RBD immunization expressed these markers at a much lower frequency (Fig. 3d–f).^25,37,38^ In addition, PEI + RBD immunization induced higher levels of lung-resident CD8^+^ T cells with CD8^+^CD69^+^CD103^−^ and CD8^+^CD69^+^CD103^+^ phenotypes, relative to PBS or RBD immunization (Supplementary Fig. 2a–c). These data indicated that in the presence of PEI adjuvant, intranasal immunization with RBD vaccine induced strong and long-lasting CD8^+^ T_RM_ cell responses in the lungs. It is reported that lung CD8^+^ T_RM_ cells are indispensable for the induction of heterosubtypic protection against respiratory pathogens.^26,30^ Therefore, CD8^+^ T_RM_ establishment after mucosal vaccination can hopefully provide cross-protection against SARS-CoV-2 infection in the lungs for a long time.

CD4^+^ T cells play vital roles in the formation of functional lung CD8^+^ T_RM_ cells during viral infection and vaccination.^28,39^ In addition, lung-resident CD4^+^ helper T cells could promote humoral response and mediate heterosubtypic protection against viral infection.^27,29^ We next investigated the effect of the adjuvanted RBD vaccine on lung CD4^+^ T-cell responses after intranasal immunization. Similarly, we observed that immunization with PEI + RBD contributed to about a threefold increase in the percentage of memory CD4^+^ T cells (CD4^+^ CD44^+^) in the lungs when compared with immunization with PBS or RBD alone (Fig. 3g, h). Memory CD4^+^ T cells from the lungs of PEI + RBD-immunized mice also showed higher expression of CD69 and/or CD103, indicating the induction of lung CD4^+^ T_RM_ cell responses (Fig. 3i–k). A great amount of the lung-resident CD4^+^ T cells elicited by PEI + RBD vaccination expressed CD69 and ~32% were CD69^+^CD103^+^ (Supplementary Fig. 2d–g). We also established t-SNE maps based on pools of CD3^+^ T cells from lung tissues of immunized mice in 1 year. We generated heatmaps to overlay the expression intensities of the surface markers CD44, CD69, and CD103. As shown in Fig. 3l, mice from the PEI + RBD group had significantly increased CD44^+^ T cells, which also presented clusters expressing CD44, CD69, and CD103 simultaneously. Therefore, intranasal immunization with PEI-adjuvanted RBD vaccine successfully established long-term mucosal immunity by inducing a balanced CD4^+^ and CD8^+^ T_RM_ cell responses in the lungs.

**Fig. 4:**
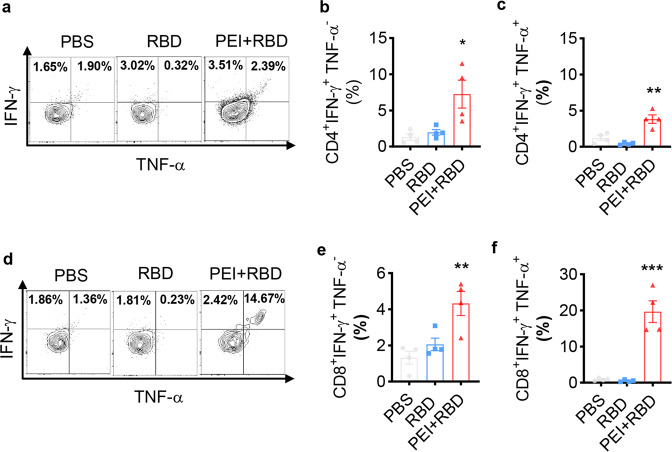
Intranasal immunization with adjuvanted RBD vaccine increases the frequency of long-lasting functional T cells in the lungs. NIH mice were intranasally immunized with PBS, RBD and PEI-adjuvanted RBD vaccine on days 0, 7, and 21. On day 365, immunized mice were sacrificed and lung tissues were harvested. The production of IFN-γ and TNF-α cytokines by lung T cells were assessed by FCM**. a** Representative FCM plots of IFN-γ and TNF-α expression in lung CD4^+^ T cells. Quantification of lung CD4^+^ IFN-γ^+^TNF-α^−^ (**b**) and CD4^+^IFN-γ^+^TNF-α^+^ (**c**) T cells. **d** Representative FCM plots of IFN-γ and TNF-α expression in lung CD8^+^ T cells. Quantification of lung CD8^+^IFN-γ^+^TNF-α^−^ (**e**) and CD8^+^IFN-γ^+^TNF-α^+^ (**f**) T cells. All the data were presented as mean ± SEM. *n* = 4. *P* values were compared to PBS group (*P < 0.05; **P < 0.01; ***P < 0.001)

We next evaluated T-cell functionality after vaccination using intracellular cytokine staining. The production of IFN-γ and TNF-α cytokines by lung T cells were determined with FCM. Mice immunized with PEI + RBD had an increased percentage of monofunctional (IFN-γ^+^TNF-α^−^) or polyfunctional (IFN-γ^+^TNF-α^+^) CD4^+^ T cells in the lungs, relative to other formulations (Fig. 4a–c). The expression of IFN-γ and/or TNF-α by lung CD8^+^ T cells were also significantly elevated after PEI + RBD immunization (Fig. 4d–f). In particular, the percentage of polyfunctional CD8^+^ T cells in the lungs exhibited about a 21-fold increase after exposure to RBD antigen and PEI adjuvant instead of PBS (Fig. 4f). Taken together, these results indicated that intranasal administration of the PEI-adjuvanted RBD vaccine can substantially enhance the magnitude, functionality, and longevity of both CD4^+^ and CD8^+^ T cells in the lungs.

### Intranasal RBD vaccine induces the formation and maturation of lung CD103^+^ DCs to promote T_RM_ generation

**Fig. 5:**
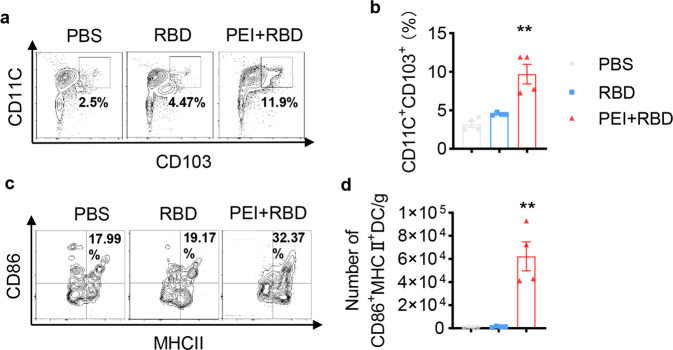
Intranasal immunization with adjuvanted RBD vaccine induces the formation and maturation of CD103^+^ DCs in the lungs. C57BL/6 mice were intranasally immunized with PBS, RBD, and PEI-adjuvanted RBD vaccine on days 0, 7, and 21. On day 28, immunized mice were sacrificed and lung tissues were harvested for the detection of CD103^+^ DCs in lung tissues with FCM. Representative FCM plots (**a**) and quantification (**b**) of CD103^+^ DCs in the lungs. **c** Representative FCM plots of CD86 and MHC II expression on lung CD103^+^ DCs. **d** Quantification of lung CD86^+^MHC-II^+^CD103^+^DCs. All the data were presented as mean ± SEM. *n* = 4. *P* values were compared to PBS group (**P < 0.01)

Eliciting robust T-cell responses requires antigen presentation by dendritic cells (DCs) which is essential for T-cell expansion and differentiation.^40^ In our previous study, we proved that PEI adjuvant could promote the maturation of bone marrow-derived DCs in vitro by upregulating maturation markers (CD40, CD86) and secreting proinflammatory cytokines (tumor necrosis factor (TNF)-α, interleukin (IL)-1β and IL-6).^32^ Local antigen presentation in nonlymphoid tissues promotes the formation of T_RM_ populations in the lungs and other tissues.^41,42^ Thus, we next assessed the effects of the PEI-adjuvanted intranasal RBD vaccine on the key antigen-presenting DC population in the lungs. Groups of C57BL/6 mice (*n* = 4) were intranasally immunized with PBS, RBD, or PEI-adjuvanted RBD on days 0, 7, and 21. On day 28, immunized mice were sacrificed and lung tissues were harvested for the analysis of DCs with FCM. The gating strategy for lung CD103^+^ DCs is shown in Supplementary Fig. 3 as previously reported.^43^ Although RBD alone had no effect on DC populations, PEI-adjuvanted RBD induced about twofold increase in the frequency of lung CD103^+^ DCs (Fig. 5a, b). In addition, immunization with PEI + RBD significantly upregulated the expression of maturation markers CD86 and major histocompatibility (MHC) class-II in lung cross-presenting CD103^+^ DCs (Fig. 5c, d).^44^

### Intranasal RBD vaccine induces the migration of activated T cells to lungs at prime and expand T cells directly in the lungs at boost

**Fig. 6:**
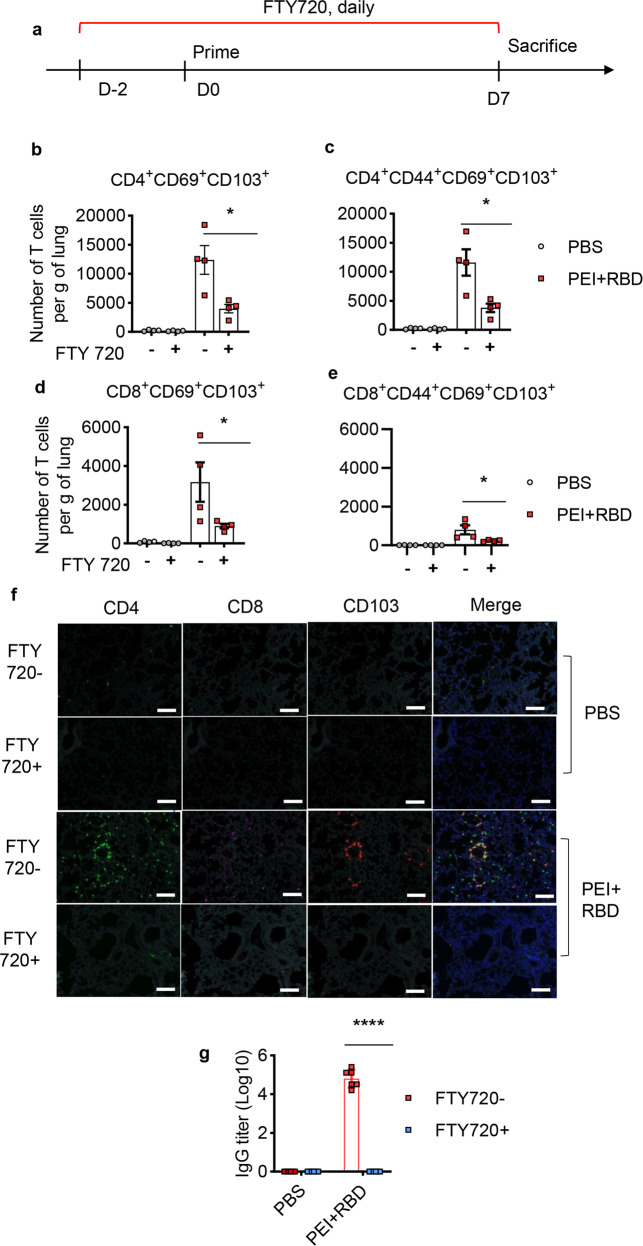
Lung T-cell responses result from T-cell migration into lungs after prime immunization with the intranasal RBD vaccine. C57BL/6 mice were primed with PBS or PEI-adjuvanted RBD vaccine in the presence or absence of FTY720 treatment. On day 7, immunized mice were sacrificed. Lung tissues and serum samples were collected for the analysis of lung T-cell responses and serum IgG levels, respectively. **a** Schedule of vaccine delivery in the presence of FTY720 during prime immunization. Quantification of lung CD4^+^ (**b**), CD4^+^CD44^+^ (**c**), CD8^+^ (**d**), and CD8^+^CD44^+^ (**e**) T cells that highly express both CD69 and CD103 after prime. **f** Immunofluorescence analysis of lung CD4^+^ and CD8^+^ T cells with elevated CD103 expression in immunized mice on 3dpi. Green, CD4; violet, CD8; red, CD103; blue, DAPI. Scale bar, 100 μm. **g** RBD-specific IgG titers in the serum after prime in the presence or absence of FTY720 were assessed with ELISA. All the data were presented as mean ± SEM. *n* = 4–5. *P* values were compared to PBS group (*P < 0.05; ****P < 0.0001)

We next explored whether the maintenance of lung T_RM_ cells is mediated by prepositioned T cells in the lungs or recruited T cells from the circulation after vaccination. Mice were treated with fingolimod (FTY720) starting two days before vaccination to block the egress of T cells from lymphoid organs (Fig. 6a).^27^ Seven days after prime immunization, lung tissues were harvested and blood samples were collected for the analysis of T-cell responses and serum IgG titers, respectively. As expected, FTY720 treatment sharply reduced the number of blood T lymphocytes, including total T cells (CD3^+^), CD4^+^ T cells, CD8^+^ T cells, memory CD4^+^ (CD4^+^CD44^+^) and CD8^+^ T cells (CD8^+^CD44^+^) (Supplementary Fig. 4a–f), regardless of vaccine immunization. This indicated the effectiveness of FTY720 to block the release of T cells into circulation. Similar to previous results, we observed that PEI-adjuvanted RBD significantly increased both the percentage and the number of lung-resident T cells as early as 7 days after prime immunization, including CD4 + , CD8 + , memory CD4 + , and memory CD8 + T cells that highly express CD69 and CD103 (Fig. 6b–e and Supplementary Fig. 5a–d). Cationic nanocarriers like PEI can prolong nasal residence time of antigens through electrostatic interactions between positively and negatively charged mucin groups.^45^ Moreover, PEI adjuvant can promote antigen uptake by and promote the maturation of antigen-presenting cells due to its particle properties.^32^ Thus, intranasal immunization with PEI-adjuvanted vaccines can elicit stronger and faster innate and adaptive immune responses than natural infection.

In the presence of FTY720, we found that the number and percentage of total CD3^+^ T cells sharply decreased even in the lungs of mice immunized with PEI + RBD (Supplementary Fig. 5e–g). Thus, it may be more appropriate to detect the variations in the absolute numbers of lung T_RM_ cells instead of the frequencies. After treatment with FTY720, only few lung-resident T cells can be detected when immunized with PEI + RBD (Fig. 6b–e). Immunofluorescence analysis of lung-resident T cells verified these findings (Fig. 6f). Moreover, FTY720 inhibited the generation of serum RBD-specific IgG antibodies at day seven after immunization with PEI + RBD (Fig. 6g). These results indicated that initial T-cell priming occurs in the lymph nodes rather than the lungs, in consistent with previous studies.^25^ We also observed a decrease in the numbers of blood T cells, including memory T cells, after immunization with PEI + RBD, comparing to PBS immunization (Supplementary Fig. 4b–f). This finding further proved that the formation of lung-resident CD4^+^ and CD8^+^ T cells requires T-cell migration from the circulation to lungs after priming.

**Fig. 7:**
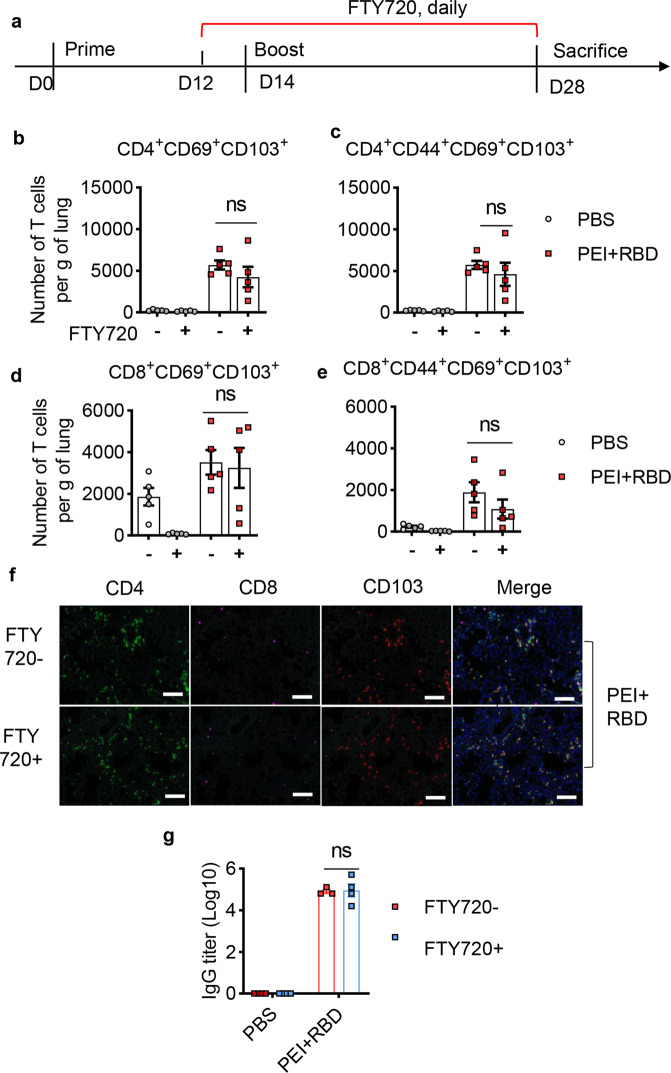
Intranasal immunization with the adjuvanted RBD vaccine directly activates T cells in the lungs at boost. NIH mice were immunized with PBS or PEI-adjuvanted RBD vaccine on days 0 and 14 in the presence or absence of FTY720 treatment. On day 28, immunized mice were sacrificed. Lung tissues and serum samples were collected for the analysis of lung T-cell responses and serum IgG levels, respectively. **a** Schedule of vaccine delivery in the presence of FTY720 during boost immunization. Quantification of lung CD4^+^ (**b**), CD4^+^CD44^+^ (**c**), CD8^+^ (**d**), and CD8^+^CD44^+^ (**e**) T cells that highly express both CD69 and CD103 after boost immunization. **f** Immunofluorescence analysis of lung CD4^+^ and CD8^+^ T cells with elevated CD103 expression in immunized mice on 3dpi. Green, CD4; violet, CD8; red, CD103; blue, DAPI. Scale bar, 100 μm. **g** RBD-specific IgG titers in the serum on day 28 in the presence or absence of FTY720 were assessed with ELISA. All the data were presented as mean ± SEM. *n* = 5. *P* values were compared to the PBS group. ns not significant

To determine where T cells were activated at boost, groups of primed mice were boosted with PBS or PEI + RBD 14 days later in the presence of FTY720 (Fig. 7a). Analysis of blood T-cell numbers proved the effectiveness of FTY720 treatment as well (data not shown). In this model, blockade of T-cell migration from the blood had no significant impact on the formation of lung-resident T cells showing CD69^+^CD103^+^ phenotype, including lung-resident CD4^+^ T cells, CD8^+^ T cells, memory CD4^+^ T cells and memory CD8^+^ T cells (Fig. 7b–e and Supplementary Fig. 6a, b). Immunofluorescence analysis also showed that FTY720 treatment did not cause any decease in lung CD4^+^ and CD8^+^ T cells that express tissue-resident marker CD103 after booster immunization (Fig. 7f). PEI + RBD immunization induced high levels of RBD-specific IgG responses in sera 14 days after booster immunization. However, FTY720 treatment had no effect on the generation of antigen-specific antibody responses if mice were primed without FTY720 treatment (Fig. 7g). Collectively, these data indicated that booster vaccination can cause local antigen presentation and self-renewal of T cells directly in the lung tissues, independent of T cells trafficking from the circulation.

### Long-term safety evaluation of the intranasal RBD vaccine in mice

**Fig. 8:**
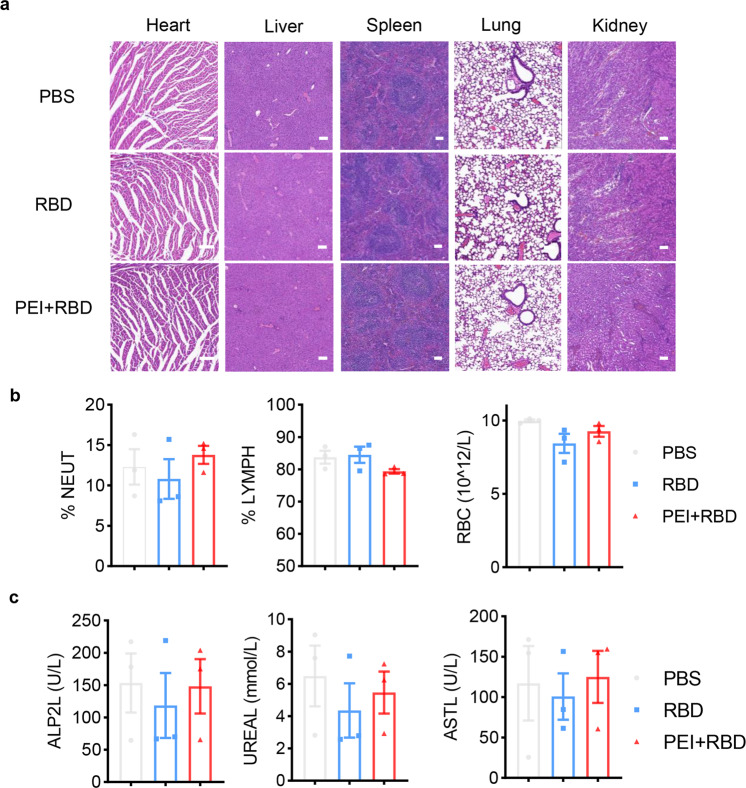
Long-term safety assessment of the adjuvanted intranasal RBD vaccine in mice. **a** H&E staining of vital organs from NIH mice immunized with PBS, RBD or PEI + RBD. Blood samples were obtained on day 365 and the peripheral blood cell counts (**b**), biochemical indexes (**c**) were examined. Scale bar, 100 μm

For safety evaluation, we monitored mice’s body weight, appearance, behavior changes, and appetite. No obvious changes were observed between normal mice and mice immunized with plain RBD or RBD + PEI. On day 365, immunized NIH mice were sacrificed and vital organs and blood samples were collected. Then, we performed H&E staining and no pathological changes were discovered in the heart, liver, spleen, lung, and kidney organs among the PBS, RBD, and PEI + RBD groups (Fig. 8a). Finally, the peripheral blood cell counts and biochemical indexes were examined, which stably remained in the normal range in immunized mice (Fig. 8b, c). These data indicated the long-term safety of our intranasal RBD vaccine in mice.

## Discussion

The continuing pandemic of COVID-19 still poses a great threat to human life and health. Early in the pandemic, the vaccine development largely focused on short-term efficacies. However, follow-up studies have reported diminished and waning immunity to the authorized COVID-19 vaccines in vaccinated individuals within several months post-immunization.^11,12,14^ Moreover, the continuous emergence of novel variants significantly compromised the effectiveness of the currently available COVID-19 vaccines.^46^ The highly mutated Omicron variant is recently of great concern owing to its high speed of transmission and possibility of immune escape.^36^ Thus, the interest of vaccine development now has shifted towards the induction of cross-reactive immunity against emerging variants and the maintenance of long-term protection. Previously, our team has developed an intranasal RBD subunit vaccine against SARS-CoV-2, which induced high levels of neutralizing antibodies in the serum and strong T-cell responses in the presence of PEI adjuvant.^32,47^ In this study, we monitored the long-term efficacy and safety of the vaccine up to day 365, and evaluated the activation of both systemic and mucosal immunity, the latter of which provides first-line defense at the entry sites of viral infection.^31^

The results indicated that the humoral immune responses induced by intranasal immunization with PEI-adjuvanted RBD vaccine retained substantially high for at least 1 year, including both neutralizing IgG antibodies in the sera and antigen-specific sIgA in the respiratory tracts (Fig. 1a, d). RBD-specific IgG subclasses, including IgG2a, IgG2b, IgG2c, and IgG1, were all relatively high, suggesting the activation of both Th1 and Th2 responses (Fig. 1c). Different mutant strains of SARS-CoV-2 covered key mutations in the spike protein region, which promotes the viral spread and reduces the effect of vaccination. In this study, the 1-year immune sera from PEI + RBD group effectively neutralized pseudovirus infection of not only WT, but also common variant strains of SARS-CoV-2, including B.1.1.7, B.1.351, P.1, P.1.617.2, Delta, and the most recent Omicron variant (Fig. 2c–f). Therefore, the intranasal RBD vaccine has the potential to induce cross-reactive immune responses that could inhibit breakthrough infections of the existing and future mutant strains of SARS-CoV-2. sIgA is a key component of mucosal immunity, which is in its dimeric form and exhibits more potent neutralizing ability than serum IgG.^24^ Similar to our observations, other studies reported that intranasal immunization with recombinant RBD vaccines induced high levels of sIgA responses in both the respiratory tracts and remote mucosal sites including genital and intestine tracts.^48^ The induction of sIgA promoted the generation of sterilizing immunity and prevented viral transmission.^22,49^ These results suggested that the intranasal RBD vaccine has the potential to prevent viral infection and shedding in both upper and lower respiratory tracts for a long time.

An attractive advantage of intranasal vaccines is the generation of T_RM_ cells in the respiratory tracts, which provides swift and robust first-line defense against mucosal pathogens.^25^ In this study, the adjuvanted RBD vaccine established and maintained strong CD4^+^ and CD8^+^ T_RM_ cell responses in the lung tissues for 1 year after intranasal immunization. This is consistent with previous researches showing that intranasal administration of a PEI-adjuvanted vaccine induced persistent antigen-specific cellular immunity in the lungs.^50^ Moreover, most studies of mucosal vaccine design focused on the induction of CD8 + T_RM_ cells, which can directly mediate cytotoxic effects on infected cells by producing cytokines/chemokines.^25,49^ Here, we found out that apart from CD8^+^ T_RM_ cells, CD4^+^ T_RM_ cell responses were also tremendously activated (Fig. 3b–k). This is important because abundant CD4^+^ T-cell responses were essential for the function of CD8 T^+^ cells and the induction of humoral immunity.^27,28^

We next explored the origins of lung T_RM_ cells and the mechanisms responsible for their durability. By using FTY720 to block peripheral T-cell recruitment, we discovered different mechanisms of lung T-cell responses induced by the adjuvanted RBD vaccine during prime and booster immunization. During the prime immunization, T cells were primed in lymph nodes and subsequently migrated to lung tissues, some of which would differentiate into lung-resident T cells that do not recirculate into the blood. On the contrary, during boosting, treatment with FTY720 failed to affect the numbers of lung-resident T cells in mice receiving PEI + RBD immunization These results indicated that the long-term maintenance of T-cell responses in the lungs may be a result of local T-cell proliferation, in consistent with previous results.^25^ It is relevant to the increase in lung CD103^+^ DCs with enhanced expression of activation marker, which could present antigens to T cells local in the lung tissues.^25^

In conclusion, we have developed an adjuvanted intranasal RBD vaccine that could induce robust and long-term immunity against both WT and multiple variant strains of SARS-CoV-2, including the most recent Omicron variant. Moreover, it induced both mucosal and systemic immunity against SARS-CoV-2 after three doses, including serum IgG antibodies, sIgA in the respiratory tracts, cellular immunity in lymph nodes,^32^ as well as CD4^+^ and CD8^+^ T_RM_ cell responses in the lungs. Finally, safety evaluation indicated the long-term tolerability of this vaccine in mice. All these results suggested that the adjuvanted intranasal RBD vaccine has the potential to be further developed for human use against the existing SARS-CoV-2 and future coronavirus pandemic.

## Materials and methods

### Materials

Recombinant RBD protein with Fc fragment (RBD-Fc) of SARS-CoV-2 and RBD-MUT (K417N, E484K, N501Y, aa: 319–541) with His fragment were purchased from Sino Biological. SARS-CoV-2 S-RBD recombinant protein was prepared by our group as previously described.^47^ Polyethyleneimine (PEI, 25k) was purchased from Sigma (USA). Goat anti-mouse IgG, IgG1, IgG2a, IgG2b, IgG2c, and IgA peroxidase conjugates were purchased from Southern Biotech. The wild-type (WT) and variant (B.1.1.7, B.1.351, P.1, P.1.617.2, and B.1.1.529) pseudoviruses of SARS-CoV-2 that highly express EGFP or luciferase were from Genomeditech. The various mutate sites were shown in previous reports.^51^ FTY720 was purchased from AbMole.

### Cells

293T cells expressing ACE2 receptor (293T/ACE2) were generated in our laboratory as previously described.^47^ The 293T/ACE2 cells were maintained in Dulbecco’s modified Eagle’s medium (DMEM, Thermo Fisher Scientific, USA) with 10% fetal bovine serum, 0.1 mg/mL streptomycin, and 100 U penicillin at 37 °C with 5% CO_2_.

### Immunization and sampling schedule

In all, 6–8 weeks of female NIH mice (Vital River, China) were purchased for immunization. The mice were divided into three groups (five mice/group) and received PBS, plain RBD, or PEI + RBD immunization by intranasal drops on day 0, 7 and 21 with 50 µL volume. Mice immunized with PBS were used as the negative control group (PBS group). Mice in RBD group were administered with 5 µg of RBD protein. In the PEI + RBD group, mice were vaccinated with 100 μg PEI plus 5 μg RBD. Blood samples and BALs were collected on day 365 and the antigen-specific antibodies in serum and BALs were detected.

In all, 6–8 weeks C57BL/6 mice (Vital River, China) were intranasally immunized with PBS or PEI + RBD with the same dose administered to NIH mice. As shown in Figs. 6a and 7a, FTY720 (20 mg/kg) was intraperitoneally injected daily for 2 days before the prime or boost immunization. The immune sera were collected 7 days after the first immunization or 14 days after the second immunization.

### Measurement of SARS-CoV-2 RBD-specific antibodies

RBD-specific serum antibodies (IgG, IgG1, IgG2a, IgG2b, IgG2c, IgA) were determined by enzyme-linked immunosorbent assay (ELISA). The flat-bottom 96-well high binding plates (Thermo Scientific, USA) were coated with 100 µL of 0.1 µg/mL RBD solution dissolved in carbonate buffer (pH 9.5) per well for about 12 h at 4 °C. We washed each well for three times using phosphate buffer saline (PBS) containing 0.05% (v/v) Tween 20 (PBS/T) and blocked the wells with 1% bovine serum albumin (BSA) at 37 °C for 1 h. 100 µL of a series of diluted sera samples or BALs were added to the plate and incubated for 1 h at 37 °C. After washing for three times, treat the plates with 100 µL of anti-mouse horseradish peroxidase conjugates including IgG, IgG1, IgG2a, IgG2b, IgG2c, and IgA diluted at 1:5000 with 1% BSA for 1 h at 37 °C. After five time washes with PBS/T, 100 µL of 3,3’,5,5’-tetramethylbiphenyldiamine (TMB) were added to the wells. Then, the reaction lasted for 10 min and was quenched with 50 µL/well-stopping solution (1.0 M H_2_SO_4_). The optical density was measured at 450 nm (A 450 nm).

### Blockade of RBD-Fc binding to ACE2 receptors

Blockade of RBD-Fc binding to cell surface ACE2 receptors was carried out by FCM. Briefly, 0.3 μg/mL of RBD-WT with Fc fragment or RBD-MUT with His fragment was incubated in the absence or presence of immune sera at different dilutions for 1 h at 37 °C. Harvest 293 T/ACE2 cells and wash them with PBS for twice. Add the incubated mixture to harvested cells (2*10^5^ /tube) and further incubate for 30 min at 4 °C. Wash cells for three times with PBS and stain them with FITC-labeled anti-human IgG Fc (Sigma-Aldrich, St. Louis, MO, USA) or anti-His (Biolegend, USA) secondary antibody for 30 min at 4 °C. After that, the mean fluorescent intensity (MFI) of each group was measured by NovoCyte Flow Cytometer (ACEA Biosciences, Inc.). The results were analyzed by NovoExpress software.

### Neutralization assay of pseudovirus infection

The pseudovirus neutralization was shown in a previous study.^47^ Briefly, immune sera were gradiently diluted by DMEM with 10% fetal bovine serum and then pre-incubated with EGFP-expressing or luciferase-expressing pseudoviruses in 96-well plates for 1 h at 37 °C, including both WT and variant (B.1.1.7, B.1.351, P.1, P.1.617.2, C.37, and Omicron) pseudoviruses. Then add 293 T/ACE2 cells at a density of 1 × 10^4^ cells per well and incubate for an additional 48 h for expression.

The efficiency of viral entry was tested with a firefly luciferase assay. In brief, the supernatants of infected cells were removed and 50 μl PBS and 50 μl lysis reagent from a luciferase kit were added. Then add luciferase substrates (Promega) to wells and detect relative light units with a multi-mode microplate reader (PerkinElmer). Use fluorescent microscopy and FCM to determine the infection efficiency of EGFP-expressing pseudoviruses in 293T/ACE2 cells.

### Measurement of immune cells in the lungs

Lungs of immunized mice were harvested and processed. Lung tissues were minced with scalpels and treated with DMEM medium containing 1% collagenase type I and 1% collagenase type IV for 1 h at 37 °C. Then the lung tissue suspension was passed through 70-mesh cell strainers and treated with red blood cell lysate to obtain single-cell suspensions. The single-cell suspensions were washed twice with PBS and resuspended in PBS containing 0.5% BSA. Cells were stained with fluorescently labeled antibodies for 30 min at 4 °C and samples were analyzed on a FCM instrument. Antibodies used to detect T_RM_s in lung tissues are as follows: anti-CD3, anti-CD4, anti-CD8, anti-CD44, anti-CD69, anti-CD103. Detection of DC cells in lung tissue used anti-CD45, anti-CD11b, anti-CD103, anti-CD11C, anti-CD86, anti-MHC II. Assessment of the functional T cells in the lungs was slightly different. Cells were stained with anti-CD3, anti-CD4, and anti-CD8 antibodies for 30 min at 4 °C, washed twice with PBS, and then fixed and permeabilized for 20 min with BD cytofix/Cytoperm (Biosciences) at 4 °C. After washing twice with BD perm/wash buffer (BD Biosciences), cells were stained with anti-IFN-γ and anti-TNF-α for 1 h at room temperature.

The expression of CD4, CD8, and CD103 in lung tissues of mice after intranasal immunization with PEI + RBD or PBS was also detected by immunofluorescence staining. Lungs were fixed in 4% paraformaldehyde for 48 h, embedded in paraffin and sectioned (3 μm). Sections were stained with the Opal 7-Color manual IHC kit (Biosciences) following the manufacturing instructions. Primary antibodies used in the procedure included rabbit anti-CD4 (Abcam, ab183685), rabbit anti-CD8α (CST, 98941), and rabbit anti-CD103 (Abcam, ab224202).

### Measurement of long-lived plasma cells in bone marrows

Bone marrow suspensions were obtained from the tibias of mice intranasally immunized with PBS, RBD, or PEI-adjuvanted RBD. The suspensions were treated with red blood cell lysis buffer and then FCM was used to detect plasma cells that producing IgG antibodies in the bone marrow. Cells in bone marrow were treated with anti-CD138 at 4 °C for 30 min. Then cells were fixed and permeabilized with the same methods used in lung T cells, followed by staining with anti-IgG antibody for 1 h at room temperature.

### Pathological evaluation of vital organs

Mice were euthanized on day 365 after the first immunization. Vital organs (lung, heart, liver, kidneys, and spleen) were isolated and fixed at 4% buffered formalin for 2 days. Then organs were embedded in paraffin, sectioned into 3-mm-thick sections and haematoxylin-eosin (H&E) staining was performed following the manufacturer’s instructions. Stained slices were scanned with an upright microscope (Nikon).

### Statistical analysis

GraphPad Prism 8 was used to perform statistical analysis. Statistical values were tested by a two-tailed unpaired Student’s *t* test or one-way ANOVA. Data were expressed as the mean ± SEM. *P* values <0.05 were considered as statistically significant (*P < 0.05; **P < 0.01; ***P < 0.001; ****P < 0.0001).

## Supplementary information

Intranasal administration of a recombinant RBD vaccine induces long-term immunity against Omicron-included SARS-CoV-2 variant

## Data Availability

All data collected in this study are available from the corresponding authors upon reasonable request.

## References

[1] Zhou P (2020). A pneumonia outbreak associated with a new coronavirus of probable bat origin. Nature.

[2] Shang J (2020). Structural basis of receptor recognition by SARS-CoV-2. Nature.

[3] Wrapp D (2020). Cryo-EM structure of the 2019-nCoV spike in the prefusion conformation. Science.

[4] Wu F (2020). A new coronavirus associated with human respiratory disease in China. Nature.

[5] Bourgonje, A. R. et al. Angiotensin-converting enzyme 2 (ACE2), SARS-CoV-2 and the pathophysiology of coronavirus disease 2019 (COVID-19). *J. Pathol*. **251**, 228–248 (2020). 10.1002/path.5471 PMC7276767 32418199

[6] Motamedi H (2021). An update review of globally reported SARS-CoV-2 vaccines in preclinical and clinical stages. Int. Immunopharmacol..

[7] Baden LR (2021). Efficacy and safety of the mRNA-1273 SARS-CoV-2 vaccine. N. Engl. J. Med..

[8] Polack FP (2020). Safety and efficacy of the BNT162b2 mRNA Covid-19 vaccine. N. Engl. J. Med..

[9] Yang S (2021). Safety and immunogenicity of a recombinant tandem-repeat dimeric RBD-based protein subunit vaccine (ZF2001) against COVID-19 in adults: two randomised, double-blind, placebo-controlled, phase 1 and 2 trials. Lancet Infect. Dis..

[10] Richmond P (2021). Safety and immunogenicity of S-Trimer (SCB-2019), a protein subunit vaccine candidate for COVID-19 in healthy adults: a phase 1, randomised, double-blind, placebo-controlled trial. Lancet.

[11] Tartof SY (2021). Effectiveness of mRNA BNT162b2 COVID-19 vaccine up to 6 months in a large integrated health system in the USA: a retrospective cohort study. Lancet.

[12] Levin EG (2021). Waning immune humoral response to BNT162b2 Covid-19 vaccine over 6 months. N. Engl. J. Med..

[13] Goldberg Y (2021). Waning Immunity after the BNT162b2 Vaccine in Israel. N. Engl. J. Med..

[14] Angel-Korman A (2022). Diminished and waning immunity to COVID-19 vaccination among hemodialysis patients in Israel: the case for a third vaccine dose. Clin. Kidney J..

[15] Seow J (2020). Longitudinal observation and decline of neutralizing antibody responses in the three months following SARS-CoV-2 infection in humans. Nat. Microbiol..

[16] Wang K (2021). Longitudinal dynamics of the neutralizing antibody response to severe acute respiratory syndrome coronavirus 2 (SARS-CoV-2) infection. Clin. Infect. Dis..

[17] Long QX (2020). Clinical and immunological assessment of asymptomatic SARS-CoV-2 infections. Nat. Med..

[18] Mlcochova P (2021). SARS-CoV-2 B.1.617.2 Delta variant replication and immune evasion. Nature.

[19] Garcia-Beltran WF (2022). mRNA-based COVID-19 vaccine boosters induce neutralizing immunity against SARS-CoV-2 Omicron variant. Cell.

[20] Harvey WT (2021). SARS-CoV-2 variants, spike mutations and immune escape. Nat. Rev. Microbiol..

[21] Kimura I (2022). The SARS-CoV-2 Lambda variant exhibits enhanced infectivity and immune resistance. Cell Rep..

[22] van Doremalen, N. et al. Intranasal ChAdOx1 nCoV-19/AZD1222 vaccination reduces viral shedding after SARS-CoV-2 D614G challenge in preclinical models. *Sci. Transl. Med*. **13**, eabh0755 (2021). 10.1126/scitranslmed.abh0755 PMC9267380 34315826

[23] Wu S (2020). A single dose of an adenovirus-vectored vaccine provides protection against SARS-CoV-2 challenge. Nat. Commun..

[24] Wang, Z. et al. Enhanced SARS-CoV-2 neutralization by dimeric IgA. *Sci. Transl. Med*. **13**, eabf1555 (2021). 10.1126/scitranslmed.abf1555 PMC7857415 33288661

[25] Rakhra, K. et al. Exploiting albumin as a mucosal vaccine chaperone for robust generation of lung-resident memory T cells. *Sci. Immunol*. **6**, eabd8003 (2021). 10.1126/sciimmunol.abd8003 PMC8279396 33741657

[26] McMaster SR, Wilson JJ, Wang H, Kohlmeier JE (2015). Airway-resident memory CD8 T cells provide antigen-specific protection against respiratory virus challenge through rapid IFN-γ production. J. Immunol..

[27] Swarnalekha, N. et al. T resident helper cells promote humoral responses in the lung. *Sci. Immunol*. **6**, eabb6808 (2021). 10.1126/sciimmunol.abb6808 PMC8063390 33419790

[28] Laidlaw BJ (2014). CD4+ T cell help guides formation of CD103+ lung-resident memory CD8+ T cells during influenza viral infection. Immunity.

[29] Zens, K. D., Chen, J. K. & Farber, D. L. Vaccine-generated lung tissue-resident memory T cells provide heterosubtypic protection to influenza infection. *JCI Insight*. **1**, (2016). 10.1172/jci.insight.85832 PMC4959801 27468427

[30] Wu T (2014). Lung-resident memory CD8 T cells (TRM) are indispensable for optimal cross-protection against pulmonary virus infection. J. Leukoc. Biol..

[31] Alu A (2022). Intranasal COVID-19 vaccines: from bench to bed. EBioMedicine.

[32] Lei H (2020). Cationic nanocarriers as potent adjuvants for recombinant S-RBD vaccine of SARS-CoV-2. Signal Transduct. Target Ther..

[33] Amanna IJ, Slifka MK (2010). Mechanisms that determine plasma cell lifespan and the duration of humoral immunity. Immunol. Rev..

[34] Gu P (2020). Polyethylenimine-coated PLGA nanoparticles-encapsulated *Angelica sinensis* polysaccharide as an adjuvant for H9N2 vaccine to improve immune responses in chickens compared to Alum and oil-based adjuvants. Vet. Microbiol..

[35] Wang Q (2014). Time course study of the antigen-specific immune response to a PLGA microparticle vaccine formulation. Biomaterials.

[36] Kannan S, Shaik Syed Ali P, Sheeza. A (2021). Omicron (B.1.1.529)—variant of concern—molecular profile and epidemiology: a mini review. Eur. Rev. Med Pharm. Sci..

[37] Sathaliyawala T (2013). Distribution and compartmentalization of human circulating and tissue-resident memory T cell subsets. Immunity.

[38] Skon CN (2013). Transcriptional downregulation of S1pr1 is required for the establishment of resident memory CD8+ T cells. Nat. Immunol..

[39] Sun JC, Bevan MJ (2003). Defective CD8 T cell memory following acute infection without CD4 T cell help. Science.

[40] Carroll EC (2016). The vaccine adjuvant chitosan promotes cellular immunity via DNA sensor cGAS-STING-dependent induction of type I interferons. Immunity.

[41] Khan TN (2016). Local antigen in nonlymphoid tissue promotes resident memory CD8+ T cell formation during viral infection. J. Exp. Med..

[42] McMaster SR (2018). Pulmonary antigen encounter regulates the establishment of tissue-resident CD8 memory T cells in the lung airways and parenchyma. Mucosal Immunol..

[43] Misharin AV (2013). Flow cytometric analysis of macrophages and dendritic cell subsets in the mouse lung. Am. J. Respir. Cell Mol. Biol..

[44] Wegmann F (2012). Polyethyleneimine is a potent mucosal adjuvant for viral glycoprotein antigens. Nat. Biotechnol..

[45] Cossette B, Kelly SH, Collier JH (2021). Intranasal subunit vaccination strategies employing nanomaterials and biomaterials. ACS Biomater. Sci. Eng..

[46] Tregoning JS (2021). Progress of the COVID-19 vaccine effort: viruses, vaccines and variants versus efficacy, effectiveness and escape. Nat. Rev. Immunol..

[47] Yang J (2020). A vaccine targeting the RBD of the S protein of SARS-CoV-2 induces protective immunity. Nature.

[48] Du Y (2021). Intranasal administration of a recombinant RBD vaccine induced protective immunity against SARS-CoV-2 in mouse. Vaccine.

[49] Hassan AO (2020). A single-dose intranasal ChAd vaccine protects upper and lower respiratory tracts against SARS-CoV-2. Cell.

[50] Bivas-Benita M (2013). Airway CD8(+) T cells induced by pulmonary DNA immunization mediate protective anti-viral immunity. Mucosal Immunol..

[51] Cascella, M. et al. *Features, Evaluation, and Treatment of Coronavirus (COVID-19)* (StatPearls Publishing © 2022, StatPearls Publishing LLC., 2022). 32150360

