# Supplementary material for: Intranasal administration of a recombinant RBD vaccine induces long-term immunity against Omicron-included SARS-CoV-2 variants
Source: Signal Transduct Target Ther. 2022 May 17;7:159. doi: 10.1038/s41392-022-01002-1 (PMC9112270; doi:10.1038/s41392-022-01002-1)
Supplement: Supplementary file 1 — Intranasal administration of a recombinant RBD vaccine induces long-term immunity against Omicron-included SARS-CoV-2 variant [file 41392_2022_1002_MOESM1_ESM.docx]

Supplementary Materials for

Intranasal administration of a recombinant RBD vaccine induces long-term immunity against Omicron-included SARS-CoV-2 variant

Hong Lei^1,#^, Aqu Alu^1,#^, Jingyun Yang^1#^, Wenyan Ren^1^ , Cai He^1^, Tianxia Lan^1^, Xuemei He, Li Yang^1,2^, Jiong Li^1,2^, Zhenling Wang^1,2^, Xiangrong Song^1,2^, Wei Wang^1,2^, Guangwen Lu^1,2*^, Xiawei Wei^1,2,*^

1. *Laboratory of Aging Research and Cancer Drug Target, State Key Laboratory of Biotherapy and Cancer Center, National Clinical Research Center for Geriatrics, West China Hospital, Sichuan University, Chengdu, 610041, China*
2. *WestVac Biopharma Co. Ltd., Chengdu, China*

# These authors contributed equally to this work

* Correspondence: Xiawei Wei ([xiaweiwei@scu.edu.cn](mailto:xiaweiwei@scu.edu.cn))，Guangwen Lu ([lugw@scu.edu.cn](mailto:lugw@scu.edu.cn))

**This PDF file includes:**

Figures. S1 to S6


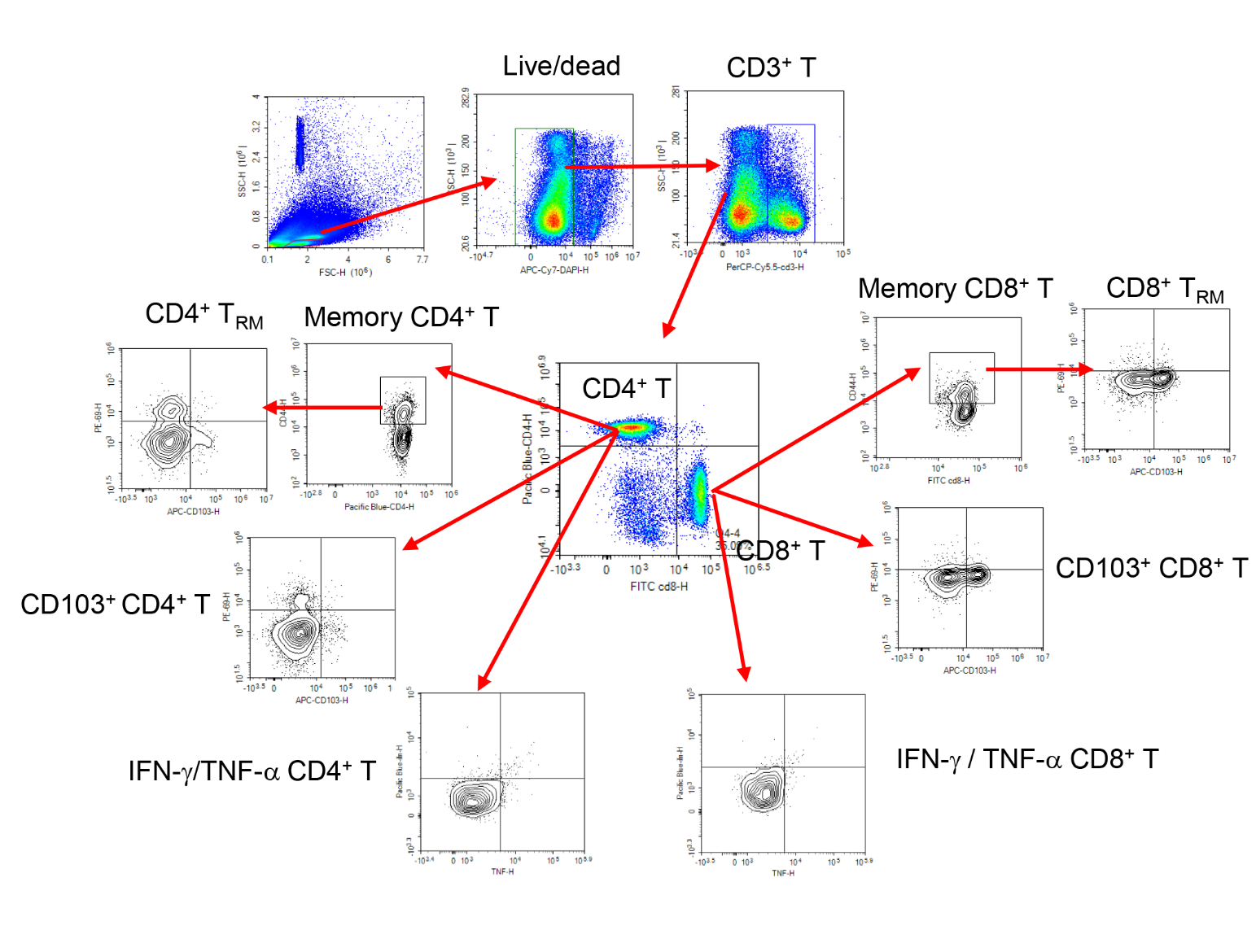


**Figure. S1** Gating strategy for tissue-resident memory T cells and functional T cells in the lungs. Depicted is the gating path in a lung sample from a mice immunized with PBS.

**
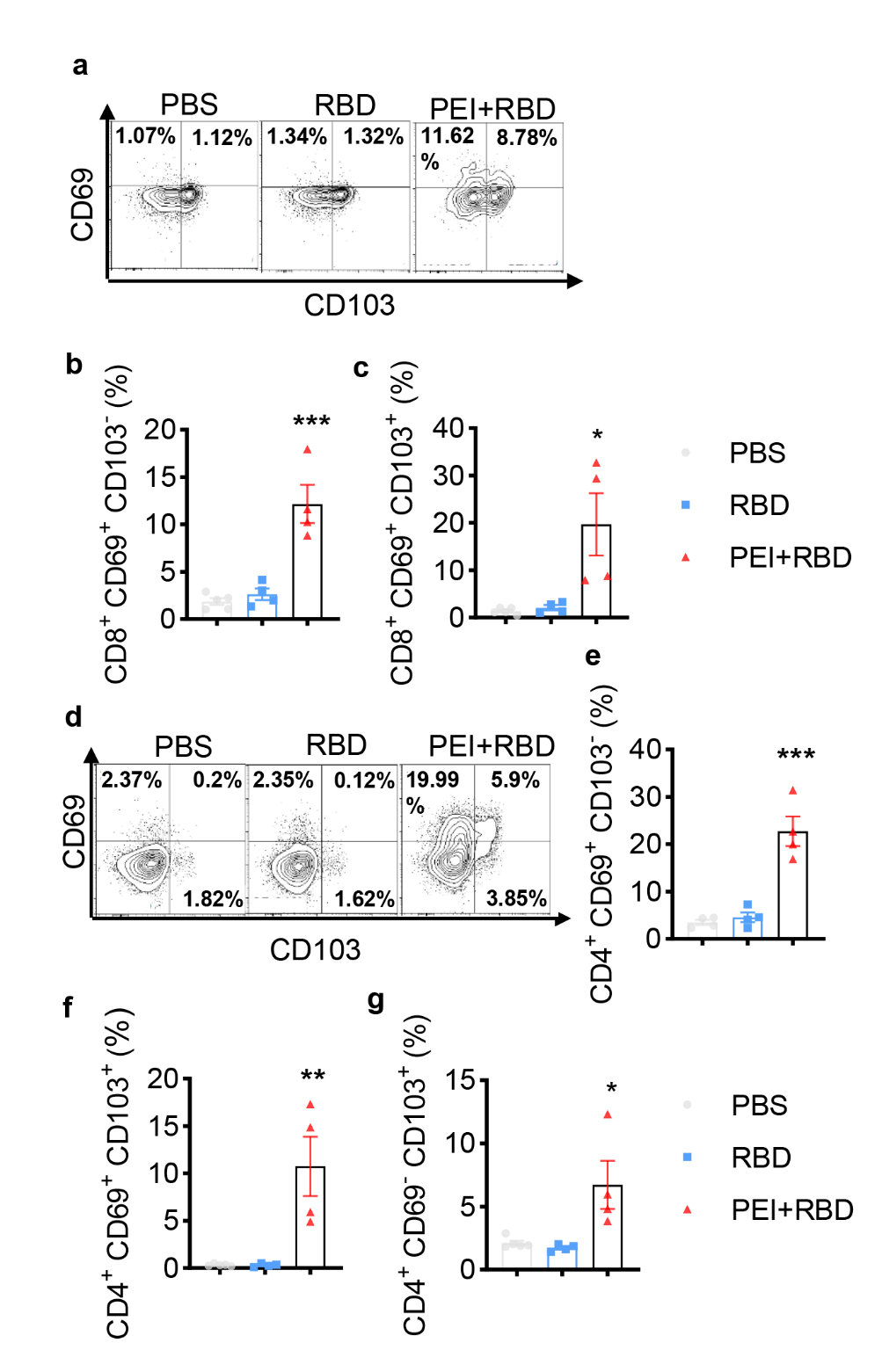
**

**Figure. S2** Intranasal immunization with adjuvanted RBD vaccine induced long-term lung-resident T cell responses. NIH mice were intranasally immunized with PBS, RBD or PEI-adjuvanted RBD on day 0, 7 and 21. Immunized mice were sacrificed in one year after prime immunization and lung tissues were harvested. Lung T cell responses were evaluated with FCM. **a** Representative FCM plots of CD69 and CD103 expression on CD4^+^ T cells in the lungs. Quantification of lung CD4^+^CD69^+^CD103^-^ (**b**), CD4^+^CD69^+^CD103^+^ (**c**) and CD4^+^CD69^-^CD103^+^ (**d**) T cells. **e** Representative FCM plots of CD69 and CD103 expression on CD8^+^ T cells in the lungs. Quantification of lung CD8^+^CD69^+^CD103^-^ (**f**) and CD8^+^CD69^+^CD103^+^ (**g**) T cells. All the data were presented as mean ± SEM. n=4. p values were compared to PBS group (*p < 0.05; **p < 0.01; ***p < 0.001).

**
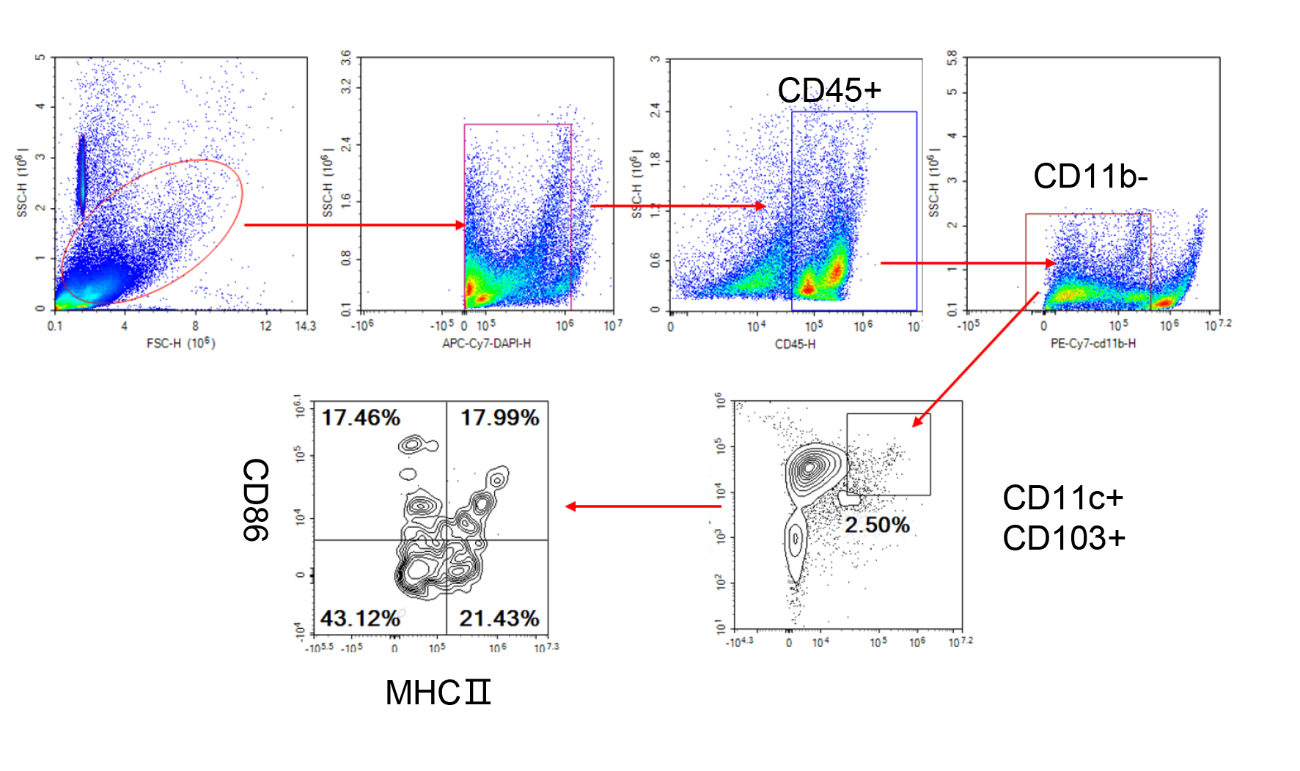
**

**Figure. S3** Gating strategy for lung CD103^+^ DCs. Depicted is the gating path in a lung sample from a PBS-immunized mice.

**
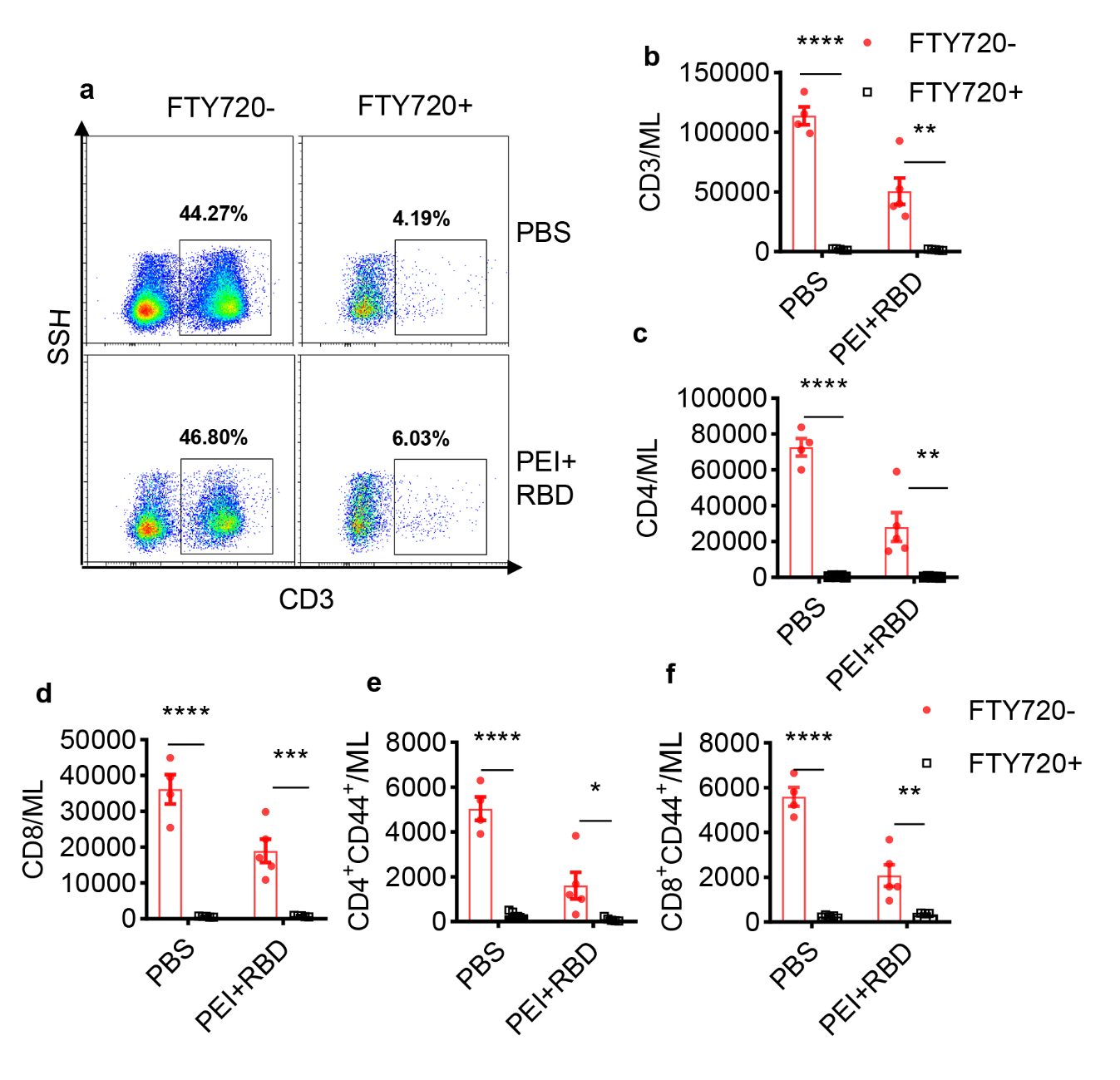
**

**Figure. S4** FTY720 treatment substantially diminished T cells in peripheral blood after immunization. **a** Representative FCM plots of CD3+ T cells in peripheral blood of mice nine days after daily FTY720 administration regardless of PEI+RBD immunization. Quantification of the numbers of CD3^+^ (**b**), CD4^+^ (**c**), CD8^+^ (**d**), CD4^+^CD44^+^ (**e**) and CD8^+^CD44^+^ (**f**) T cells in peripheral blood of mice nine days after daily FTY720 administration regardless of PEI+RBD immunization. All the data were presented as mean ± SEM. n=4~5. p values were compared to PBS group (*p < 0.05; **p < 0.01; ***p < 0.001, ****p < 0.0001).


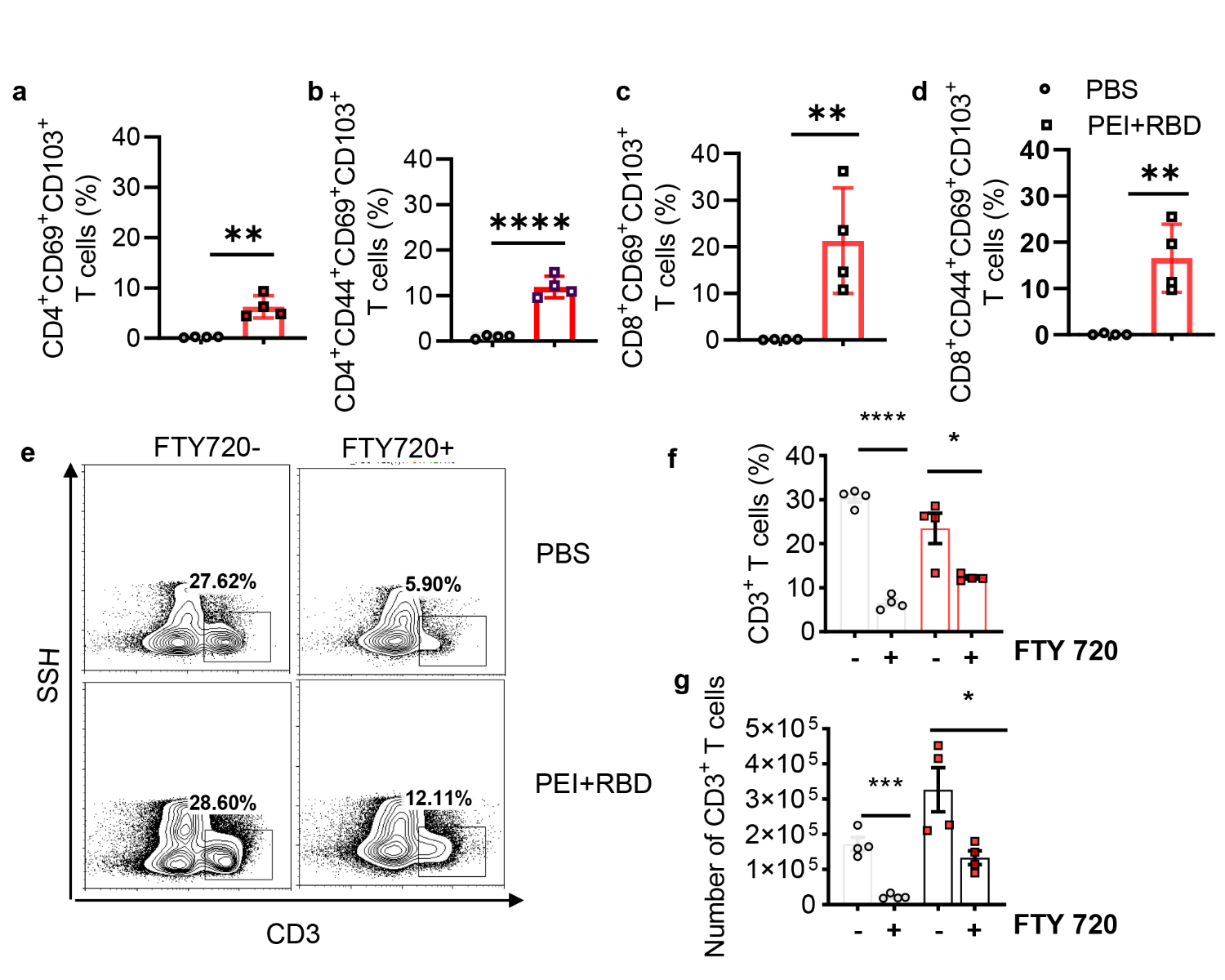


**Figure. S5** Lung T cell responses are resulted from T cell migration into lungs after prime immunization with the intranasal RBD vaccine. Mice were primed with PBS or PEI-adjuvanted RBD vaccine in the presence or absence of FTY720 treatment. On day seven, immunized mice were sacrificed. Lung tissues were collected for the analysis of lung T cell responses. Percentage of lung CD4^+^CD69^+^CD103^+^ (**a**), CD4^+^CD44^+^CD69^+^CD103^+^ (**b**), CD8^+^ CD69^+^CD103^+^ (**c**) and CD8^+^CD44^+^ CD69^+^CD103^+^ (d) T cells. **e** Representative FCM plots of CD3^+^ T cells in lung of mice nine days after daily FTY720 administration regardless of PEI+RBD immunization. Quantification of the numbers (f) or percentage (g) of CD3^+^ T cells in lung of mice.


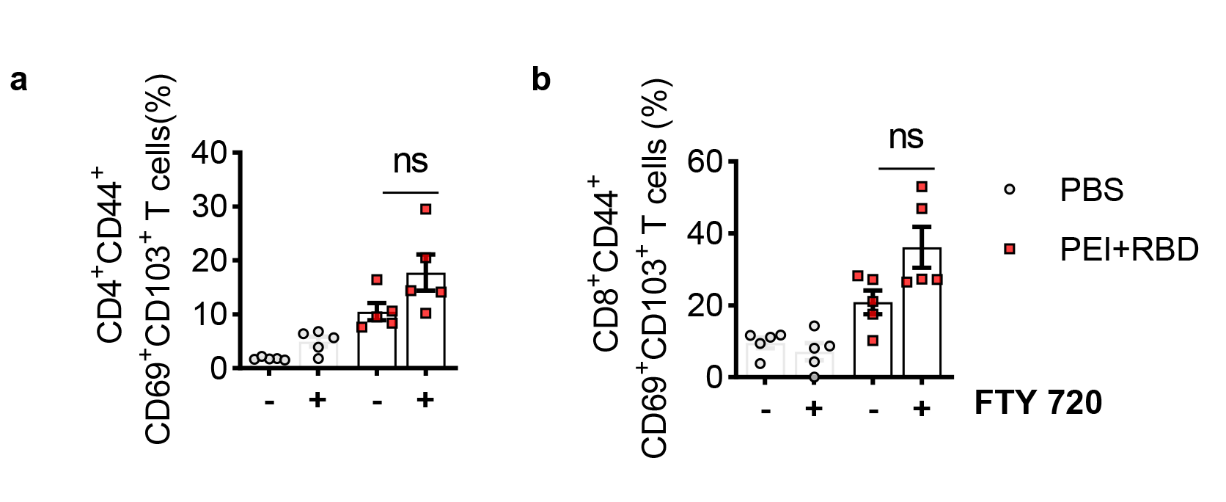


**Figure. S6** Intranasal immunization with the adjuvanted RBD vaccine directly activates CD8 T cells in the lungs at boost. Mice were immunized with PBS or PEI-adjuvanted RBD vaccine on day 0 and 14 in the presence or absence of FTY720 treatment. On day 28, immunized mice were sacrificed. Lung tissues were collected for the analysis of lung T cell responses. Quantification of percentage of lung CD4^+^CD44^+^CD69^+^CD103^+^ (**a**) and CD8^+^CD44^+^CD69^+^CD103^+^ (**b**) T cells after boost immunization.
